# Surgical resection of pediatric PRETEXT III and IV hepatoblastoma: A retrospective study investigating the need for preoperative chemotherapy

**DOI:** 10.3389/fped.2022.878095

**Published:** 2022-12-01

**Authors:** Xiongwei Wu, Jianyong Wang, Yuhe Duan, Yusheng Liu, Yao Liu, Xin Chen, Nan Xia, Qian Dong

**Affiliations:** ^1^ Department of Pediatric Surgery, Affiliated Hospital of Qingdao University, Qingdao, China; ^2^ Shandong Key Laboratory of Digital Medicine and Computer Assisted Surgery, Affiliated Hospital of Qingdao University, Qingdao, China; ^3^ Department of Pediatrics, Yantai Yuhuangding Hospital, Shandong, China

**Keywords:** hepatoblastoma (HB), surgery, preoperative chemotherapy, surgical resection, liver vasculature

## Abstract

**Objective:**

This study analyzed the feasibility of upfront surgical resection for pediatric PRETEXT III and IV hepatoblastoma (HB).

**Summary Background Data:**

Neoadjuvant chemotherapy is recommended for patients with PRETEXT III and IV HB to obtain a chance of curative surgery. However, chemotherapy can cause toxic side effects and adverse outcomes, and the PRETEXT staging system may overstage the patients. Therefore, whether preoperative chemotherapy is necessary for HB patients remains unclear.

**Methods:**

The clinical data of 37 children who underwent surgical resection for PRETEXT III and IV HB at our hospital were obtained retrospectively. Patients were divided into the neoadjuvant chemotherapy group (NCG; *n* = 19) and the routine surgery group (RSG; *n* = 18). Clinicopathologic characteristics, treatment regimens, and outcomes were compared between the groups.

**Results:**

The RSG had a lower incidence of portal vein involvement than the NCG (*p* < 0.002). The estimated 3-year event-free survival rates were similar (RSG: 89 ± 0.7% and NCG: 79 ± 0.9%, *p* = 0.3923). The RSG underwent fewer courses of chemotherapy than the NCG (five vs. six; *p* < 0.001). Furthermore, the RSG had lower incidences of febrile neutropenia, myelosuppression, and gastrointestinal reactions (all *p* < 0.05). The severity of surgery-related complications did not differ significantly.

**Conclusion:**

Upfront surgical resection in children with PRETEXT III and IV HB is safe and feasible, and reduces the total number of courses and side effects of chemotherapy. The degree of vascular involvement is the most important consideration when evaluating resectability during diagnosis.

## Introduction

Hepatoblastoma (HB) is the most common pediatric primary liver malignancy, accounting for approximately 90% of liver tumors in children (1). Radical surgery remains the cornerstone of treatment for hepatoblastoma (2). The PRE-treatment Tumor EXTension (PRETEXT) staging system designed by the International Childhood Liver Tumor Strategy Group (SIOPEL), combined with detailed annotation factors, can guide the indications for surgical resection and has become an internationally recognized standard (3–5). Previously, liver transplantation for hepatoblastoma included unifocal POST-TEXT IV (PRETEXT classification following chemotherapy) and/or POST-TEXT III or IV tumors with persistent widespread multifocality or major vessel involvement (6). Using neoadjuvant chemotherapy, studies have reported that extensive liver resection for POST-TEXT III and IV hepatoblastoma appears to have a comparable overall survival (OS) rate compared with liver transplantation when combined with chemotherapy (7, 8). Interestingly, Fuchs et al. performed extensive liver resection in 27 patients with POST-TEXT III and IV hepatoblastoma, and the 5-year OS rate was 80.7% (9). This aggressive surgical resection may mitigate the need for orthotopic liver transplantation in selective advanced cases.

Although chemotherapy is beneficial to children with HB, it can also cause toxic side effects and adverse outcomes (10). Furthermore, only a few studies have shown that the PRETEXT staging system tends to overstage patients (11), thus, delaying surgery and causing overtreatment. Additionally, a biopsy is required for a clear pathological diagnosis; however, chemotherapy is occasionally administered to patients whose pathological type cannot be determined (12) and studies have reported that preoperative chemotherapy can transform an originally resectable tumor into an unresectable type (13). Therefore, it is controversial whether preoperative chemotherapy is necessary for patients with HB (14, 15). For the 49 patients with low-risk HB in the AHEP0731 trial, chemotherapy was unnecessary when the tumor was completely resectable during diagnosis. It is worth noting that this included one patient with PRETEXT IV HB and two with PRETEXT III HB, although the authors did not specifically discuss the cases of these three patients (16). Only a few reports have focused on the feasibility of upfront surgical resection in children with PRETEXT stage III and IV HB exist.

Thus, this study retrospectively analyzed the clinical data of children with PRETEXT III and IV HB who underwent surgical resection in our hospital and explored whether preoperative chemotherapy is necessary for such children.

## Materials and methods

### Patients

We performed a retrospective review of children with HB who underwent surgical resection at the Affiliated Hospital of Qingdao University between January 2008 and December 2018. The inclusion criteria were as follows: (1) age <14 years, (2) postoperative pathologically confirmed PRETEXT III or IV HB, and (3) complete clinicopathological data. The exclusion criteria were as follows: (1) distant metastasis at diagnosis; presence of (2) contiguous extrahepatic tumor (3) multifocal tumor (4) history of any other congenital disease.

This study was approved by the Ethics Committee of the Affiliated Hospital of Qingdao University and was conducted following the principles of the Declaration of Helsinki. Written informed consent was obtained from the patients or their guardians.

### Clinical data collection

The children were divided into the neoadjuvant chemotherapy group (NCG) and the routine surgery group (RSG) based on the administration of neoadjuvant chemotherapy. Clinicopathologic characteristics, treatment regimens, and outcomes were compared between the groups. Postoperative complications were assessed using the Clavien–Dindo classification system. If the patient had more than one complication, the most serious complication was recorded (17). Patients were staged for risk classification using the Evans surgical staging guidelines (18), wherein Stage I is defined as complete resection with microscopically negative margins, whereas Stage II is defined as complete resection with microscopic residual disease at the resection margins. The proximity of the tumor to the major hepatic veins, inferior vena cava, or bifurcation of the portal vein was centrally reviewed and coded (16) and the detailed definitions are presented in Table 1. All patients were followed up until December 31, 2021.

**Table 1:** Grading the proximity of the tumor to the major liver vasculature.

Code	Definition
Negative	Tumor >1 cm from the vessels
0	Tumor within 1 cm of the vessels
1	Tumor touching the vessels
2	Tumor distorting, displacing, or encasing the vessels
3	Radiographically identifiable tumor thrombus within the lumen of the major vessels

### Preoperative evaluation and chemotherapy

**Figure 1:**
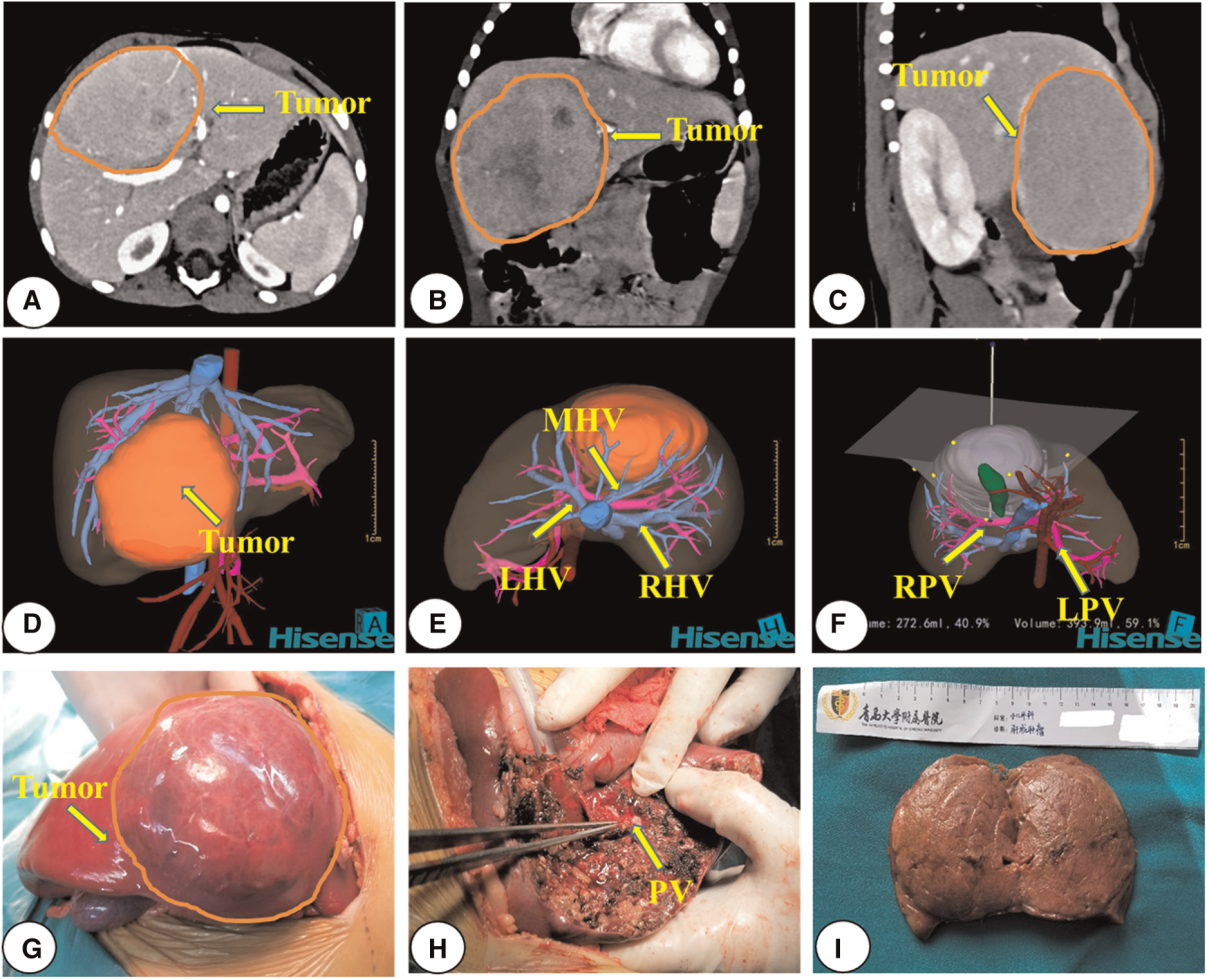
Representative preoperative assessment and surgical status of a patient with hepatoblastoma who underwent upfront surgery. (**A–C**) CT images; (**D–F**) 3D reconstruction images; (**G–I**) intraoperative observations. CT, computed tomography; 3D, three-dimensional; RPV, right portal vein; LPV, left portal vein; PV, portal vein; RHV, right hepatic vein; LHV, left hepatic vein; MHV, middle hepatic vein; AA, Abdominal Aorta.

**Figure 2:**
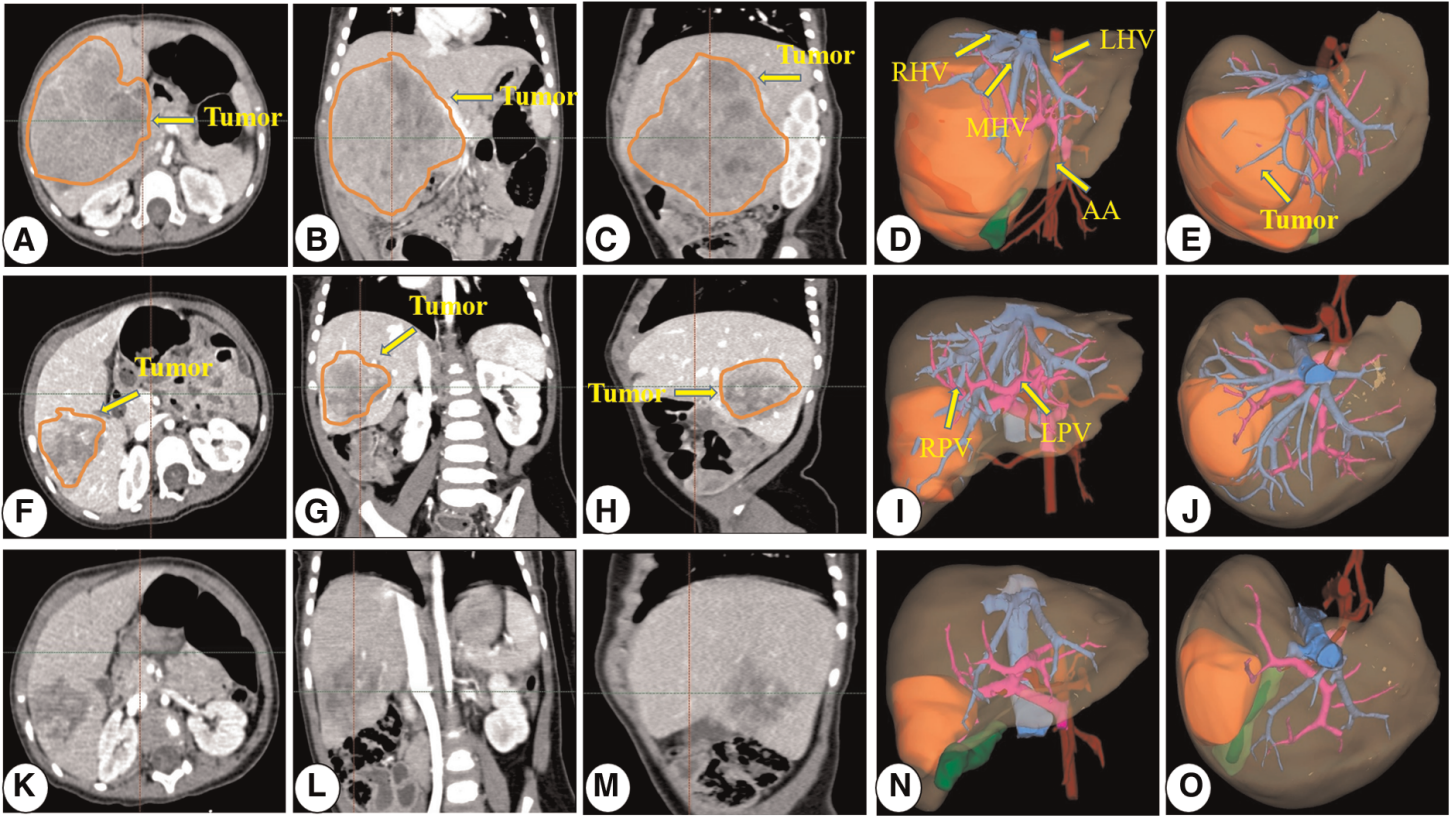
Representative preoperative chemotherapy assessment of a patient who underwent neoadjuvant chemotherapy before surgical resection. (**A–E**) CT images and 3D reconstruction images at diagnosis; (**E–J**) CT images and 3D reconstruction images after two courses of preoperative chemotherapy; (**K–O**) CT images and 3D reconstruction images after three courses of preoperative chemotherapy. CT, computed tomography; 3D, three-dimensional; RPV, right portal vein; LPV, left portal vein; PV, portal vein; RHV, right hepatic vein; LHV, left hepatic vein; MHV, middle hepatic vein; AA, abdominal aorta.

Each child with HB underwent enhanced abdominal computed tomography and was evaluated using the Hisense computer-assisted surgery system (Hisense CAS, version CAS-V3.01.4775) three-dimensional (3D) reconstruction (19, 20) during diagnosis. The surgical team developed the treatment plan by considering the tumor location, the relationship between tumors and major vessels, and outcomes of virtual hepatectomy based on 3D reconstruction. The criterion for judging surgical resection with 3D reconstruction was a residual liver volume >30% after surgical resection of the tumor, and the absence of symptoms of ischemia or blood stasis in the resected liver. Upfront surgical resection was performed directly on children with HB with resectable tumors (i.e., the RSG; Figure 1). For those with unresectable tumors during diagnosis (i.e., the NCG), resectability was reassessed after two to four courses of chemotherapy (Figure 2). All patients underwent routine postoperative chemotherapy after surgery. Implementation of our chemotherapy regimens followed the Multidisciplinary Treatment Guideline for Chinese Children with Hepatoblastoma developed by the Chinese Children's Cancer Group, using SIOPEL and COG as references (21, 22).

### Surgical procedure

According to the preoperative three-dimensional images of the computer-aided surgery system and the virtual liver resection plan and verification, the location and size of the tumor and its relationship with the surrounding organs and blood vessels were routinely explored in the abdomen. The first, second, and third hepatic portals were carefully dissected for hepatectomy, and the hepatic artery, hepatic vein, short hepatic vein, and bile duct were carefully handled during the operation to prevent postoperative bile leakage and bleeding. When dividing the liver parenchyma and tumor, we used a cavitron ultrasonic surgical aspirator to cut the liver parenchyma and ligate and cut off the blood vessels and bile ducts. To reduce intraoperative bleeding, the first hepatic hilum can be blocked, but this procedure should last <20 min.

### Statistical analysis

Statistical analysis was performed using SPSS 26.0 (IBM Corp., Armonk, NY, USA), and data were plotted using GraphPad Prism 9 (GraphPad Software, San Diego, CA, USA). Variables are expressed as means ± SD, medians [interquartile ranges (IQRs)], or numbers (percentages), according to the type of variable. Comparisons between the two groups were performed using Student's *t*-test, Mann–Whitney *U* test, *χ*^2^ test, or Fisher's exact test, as appropriate.

Event-free survival (EFS) was defined as the time from the date of diagnosis to the date of one of the following events: progression, recurrence, death, or complete remission. Kaplan–Meier survival curves were used to estimate the EFS probabilities, and differences were calculated using the log-rank test. Differences were considered statistically significant at *p* < 0.05.

## Results

A total of 37 children with HB were included in this study. Patient characteristics are presented in Table 2. The RSG included 18 patients (13 male and five female patients), and the NCG included 19 patients (12 male and seven female patients), with a median age of 11.5 (IQR, 7.75–28.5) and 15.0 (IQR, 12.0–24.0) months, respectively. No statistical difference was observed between the two groups in age, sex, PRETEXT stage, involvement of the inferior vena cava, or AFP level during diagnosis (all *p* > 0.05). The RSG had a lower incidence of portal vein involvement than the NCG (*p* = 0.044).

**Table 2:** Clinicopathological data of the two groups.

Various	RSG (*n* = 18)	NCG (*n* = 19)	*p*
Age^a^, months, median (IQR)	11.5 (7.75–28.5)	15.0 (12.0–24.0)	0.626
Sex^b^			0.728
Male	13 (72.2%)	12 (63.2%)	
Female	5 (27.8%)	7 (36.8%)	
PRETEXT stage^b^			0.746
III	10 (55.6%)	9 (47.4%)	
IV	8 (44.4%)	10 (52.6%)	
Vascular invasion^b^			
V			0.393
V negative	5 (27.8%)	1 (5.3%)	
V0	4 (22.2%)	6 (31.6%)	
V1	4 (22.2%)	4 (21.1%)	
V2	5 (27.8%)	7 (36.8%)	
V3	0	1 (5.3%)	
P			0.044
P negative	2 (11.1%)	0	
P0	3 (16.7%))	0	
P1	7 (38.9%)	6 (31.6%)	
P2	6 (33.3%)	13 (68.4%)	
P3	0	0	
AFP^b^, ng/ml			0.486
<100	1 (5.6%)	0	
≥100	17 (94.4%)	19 (100%)	

IQR, interquartile range.

^a^ Data are presented as medians (*p*-values were derived from the Mann–Whitney *U* test).

^b^ Data are presented as *n* (*p*-values were derived from the Fisher's exact tests, in percent).

Patients in the RSG received fewer courses of chemotherapy than those in the NCG [5 (IQR, 4.75–5.25) vs. 6 (IQR, 6–8), *p* < 0.001]. Furthermore, the RSG had lower incidences of febrile neutropenia (61.6% vs. 94.7%, *p* = 0.019), bleeding (5.6% vs. 84.2%, *p* < 0.001), and anemia (5.6% vs. 89.5%, *p* < 0.001) than the NCG. The incidence of gastrointestinal disorders was also lower in the RSG than in the NCG (66.7% vs. 94.7%, *p* = 0.042). However, no statistically significant differences were observed between the two groups in the incidences of other chemotherapy complications, including electrolyte disorders; ototoxicity; convulsions; and heart, liver, and kidney dysfunction (Table 3).

**Table 3:** Comparison of chemotherapy complications between the two groups.

Complications	RSG	NCG	*p*
Number of courses of chemotherapy^a^, median (IQR)	5 (4.75–5.25)	6 (6–8)	<0.001
Febrile neutropenia^b^	11 (61.1%)	18 (94.7%)	0.019
Intestinal function disorder^b^	12 (66.7%)	18 (94.7%)	0.042
Electrolyte disturbance^b^	4 (22.2%)	6 (31.6%)	0.714
Anemia^b^	1 (5.6%)	17 (89.5%)	<0.001
Hemorrhage^b^	1 (5.6%)	16 (84.2%)	<0.001
Abnormal liver function^b^	6 (33.3%)	12 (63.2%)	0.103
Myocardial injury^b^	2 (11.1%)	4 (21.1%)	0.660
Ototoxicity^b^	2 (11.1%)	5 (26.3%)	0.405
Convulsions^b^	2 (11.1%)	3 (15.8%)	>0.99
Renal toxicity^b^	0	2 (10.5%)	0.486

IQR, interquartile range.

^a^ Data are presented as medians (*p*-values were derived from the Mann–Whitney *U* test).

^b^ Data are presented as *n* (*p*-values were derived from the Fisher's exact tests, in percent).

All patients with HB underwent surgery with R0 resection. No statistical difference between the two groups in the average operation time, intraoperative blood transfusion, severity of Clavien–Dindo complications, or Evans surgical stage was observed (all *p* > 0.05). A comparison of surgery-related parameters in the two groups is presented in Table 4.

**Table 4:** Comparison of surgery-related parameters between the two groups.

Operation parameters	RSG (*n* = 18)	NCG (*n* = 19)	*p*
Operative time^a^ (min), mean ± SD	183.83 ± 45.51	197.37 ± 36.69	0.325
Intraoperative blood transfusion^a^ (ml/kg)	13.03 ± 6.35	13.37 ± 5.32	0.895
Postoperative Clavien–Dindo complication stage^b^			0.279
I	8 (44.4%)	3 (15.8%)	
II	8 (44.4%)	13 (68.4%)	
III	1 (5.6%)	1 (5.3%)	
IV	1 (5.6%)	2 (10.5%)	
Evans surgical stage^b^			0.66
I	16 (88.9%)	15 (78.9%)	
II	2 (11.1%)	4 (21.1%)	

SD, standard deviation.

^a^ Data are presented as mean (*p*-values were derived from the Student's *t*-test).

^b^ Data are presented as *n* (*p*-values were derived from the Fisher's exact tests, in percent).

**Figure 3:**
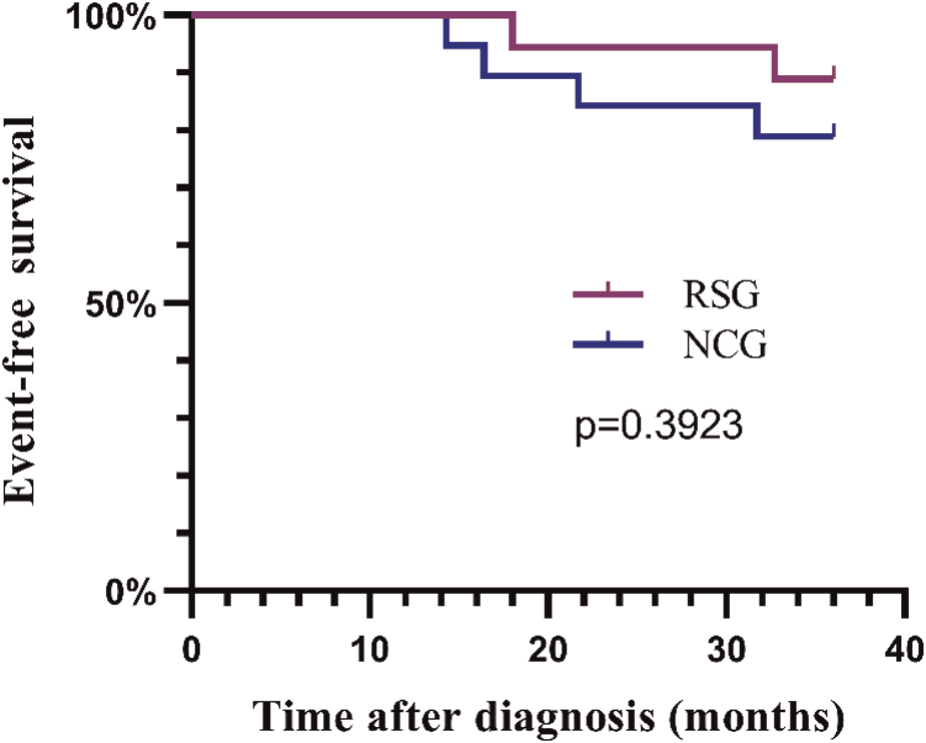
The 3-year event-free survival curves for the RSG and NCG. RSG, routine surgery group; NCG, neoadjuvant chemotherapy group.

The median follow-up period was 49 months. During the follow-up period, three of the 19 children in the NCG developed recurrence and one developed lung metastasis; no deaths were reported. Two of 18 children in the RSG died, one of whom died 6 months after the recurrence. The estimated 3-year EFS rate in the RSG was similar to that in the NCG (89 ± 0.7% and 79 ± 0.9%, respectively; *p* = 0.3923; Figure 3).

## Discussion

Preoperative chemotherapy results in good outcomes in children with HB; however, it also increases the risk of chemotherapy complications. Studies have reported that the prognosis of children with PRETEXT I and II HB undergoing surgical resection without preoperative treatment is not worse than that of those who receive preoperative chemotherapy (23). Additionally, upfront surgical resection in patients with PRETEXT I and II HB can reduce the number of courses and complications of chemotherapy (16). However, only a few studies on upfront resections in patients with PRETEXT III or IV HB have been reported. We retrospectively analyzed the treatment outcomes of children with PRETEXT III and IV HB at our center and observed that upfront surgical resection is safe and feasible in these patients, bringing new insights into the timing of surgery for children with PRETEXT III and IV HB.

A previous study observed that the 3-year EFS rate for patients with PRETEXT III and IV HB after neoadjuvant chemotherapy and resection was 75.7% (24). Another study reported that the 3-year EFS rate of patients with PRETEXT IV HB who underwent resection after neoadjuvant chemotherapy was 76% (25). These results are consistent with the 3-year EFS rate of 79% in the NCG of our study. However, the 3-year EFS rate in the RSG was 89%, indicating that upfront surgical resection in patients with PRETEXT III and IV HB may result in improved outcomes.

We observed that upfront surgical resection in patients with PRETEXT III and IV HB significantly reduces the total number of chemotherapy sessions. Multiple toxic effects of chemotherapeutic drugs administered for HB are observed, including myelosuppression, infection, gastrointestinal reactions, liver and kidney toxicity, and ototoxicity (26). Myelosuppression with infection is the most common complication of chemotherapy (16), and it can cause septic shock and death in severe cases. In our study, the incidences of febrile neutropenia, anemia, and bleeding were significantly lower in the RSG than in the NCG. Platinum-based chemotherapy is essential for HB treatment (27), and its most common side effect is ototoxicity (28), which can cause permanent damage in children (29) and is related to the platinum accumulation. In our study, the incidence of ototoxicity was 11.1% and 26.3% in the RSG and NCG, respectively. However, no significant difference in the incidence of ototoxicity between the two groups was observed, probably because of the small sample size. In addition, the incidence of gastrointestinal reactions was also significantly lower in the RSG than in the NCG. Therefore, the advantage of this treatment strategy is that it reduces acute and long-term drug toxicity and medical expenses by lowering the course of chemotherapy and the accumulation of these drugs.

PRETEXT III and IV HB tumors tend to be larger and compress or invade the surrounding blood vessels, causing the tumor margins to be <1 cm away from important blood vessels and making resection more difficult. In this study, most of the tumors in the RSG were large and were <1 cm from the major blood vessels. However, the incidences of intraoperative and postoperative complications were not higher in the RSG than in the NCG, indicating that upfront surgical resection is safe. In a previous study by our group, an obvious fibrous capsule around the HB was observed, which blocked the local tumor invasion; thus, even if the surgical margin is narrow (<1 cm), the tumor can be surgically removed safely and completely (20). In addition, studies have reported that the recurrence rates of tumors with surgical margins <1 cm and those with surgical margins >1 cm were the same (30). When the surgical margin is <1 cm, the patient may have microscopic positive surgical margins; however, our research reported no significant differences in the microscopic positive surgical margin rates between the two groups. Therefore, even if the surgical margin is microscopically positive, it does not affect the prognosis (31, 32).

Our study results demonstrated that upfront resection was associated with a lower incidence of portal vein involvement. Vascular invasion is considered a risk factor affecting the prognosis of children with HB (33–36) and is also an important indicator of resectability (37). Although the latest PRETEXT system considers vascular invasion (4), it tends to overstage tumors (11). Distinguishing between PRETEXT II and PRETEXT III can be especially difficult because the question of tumor compression vs. tumor invasion of the adjacent liver parenchyma exists (4). Hisense CAS 3D reconstruction indicated PRETEXT overstaging in three patients in the RSG, which was confirmed during surgery. Therefore, we suggest that the degree of vascular involvement should be the most important reference factor for assessing the surgical resectability of PRETEXT III and IV HB. However, this does require a computer-assisted surgery system for 3D reconstruction and an experienced surgeon to conduct the preoperative evaluation.

However, our study had a few limitations. First, this study was a retrospective analysis of a small sample from a single medical institution; therefore, a potential selection bias may have been present. Second, our study lacked external verification. Finally, because of the short follow-up period, we could only analyze the EFS at 3 years, although we will continue to follow these patients. We look forward to conducting a large-sample, multicenter, prospective collaborative study in the future to support the conclusions of our research. In summary, this study confirmed that upfront surgical resection is safe and feasible for children with PRETEXT III and IV HB, and it can reduce the course and side effects of chemotherapy. The degree of vascular involvement is the most important consideration when assessing resectability. Our study provides new insights into the timing of surgery for children with PRETEXT III and IV HB.

## Data Availability

The original contributions presented in the study are included in the article/Supplementary Material, further inquiries can be directed to the corresponding author/s.

## References

[1] Darbari A Sabin KM Shapiro CN Schwarz KB. Epidemiology of primary hepatic malignancies in U.S. Children. Hepatology. (2003) 38(3):560–6. 10.1053/jhep.2003.50375 12939582

[2] Malogolowkin MH Katzenstein HM Meyers RL Krailo MD Rowland JM Haas J Complete surgical resection is curative for children with hepatoblastoma with pure fetal histology: a report from the children’s oncology group. J Clin Oncol. (2011) 29(24):3301–6. 10.1200/JCO.2010.29.3837 21768450 PMC3158601

[3] Roebuck DJ Aronson D Clapuyt P Czauderna P de Ville de Goyet J Gauthier F 2005 Pretext: a revised staging system for primary malignant liver tumours of childhood developed by the siopel group. Pediatr Radiol. (2007) 37(2):123–32; quiz 249–50. 10.1007/s00247-006-0361-5 17186233 PMC1805044

[4] Towbin AJ Meyers RL Woodley H Miyazaki O Weldon CB Morland B 2017 Pretext: radiologic staging system for primary hepatic malignancies of childhood revised for the paediatric hepatic international tumour trial (phitt). Pediatr Radiol. (2018) 48(4):536–54. 10.1007/s00247-018-4078-z 29427028

[5] Meyers RL Katzenstein HM Malogolowkin MH. Predictive value of staging systems in hepatoblastoma. J Clin Oncol. (2007) 25(6):737; author reply -8. 10.1200/JCO.2006.06.4915 17308285

[6] Trobaugh-Lotrario AD Meyers RL Tiao GM Feusner JH. Pediatric liver transplantation for hepatoblastoma. Transl Gastroenterol Hepatol. (2016) 1:44. 10.21037/tgh.2016.04.01 28138611 PMC5244811

[7] Fonseca A Gupta A Shaikh F Ramphal R Ng V McGilvray I Extreme hepatic resections for the treatment of advanced hepatoblastoma: are planned close margins an acceptable approach? Pediatr Blood Cancer. (2008) 65(2):e26779. 10.1002/pbc.26820 28921939

[8] Lautz TB Ben-Ami T Tantemsapya N Gosiengfiao Y Superina RA. Successful nontransplant resection of post-text III and IV hepatoblastoma. Cancer. (2011) 117(9):1976–83. 10.1002/cncr.25722 21509775

[9] El-Gendi A Fadel S El-Shafei M Shawky A. Avoiding liver transplantation in post-treatment extent of disease III and IV hepatoblastoma. Pediatr Int. (2018) 60(9):862–8. 10.1111/ped.13634 29906299

[10] Malogolowkin MH Katzenstein H Krailo MD Chen Z Bowman L Reynolds M Intensified platinum therapy is an ineffective strategy for improving outcome in pediatric patients with advanced hepatoblastoma. J Clin Oncol. (2006) 24(18):2879–84. 10.1200/JCO.2005.02.6013 16782927

[11] Aronson DC Schnater JM Staalman CR Weverling GJ Plaschkes J Perilongo G Predictive value of the pretreatment extent of disease system in hepatoblastoma: results from the international society of pediatric oncology liver tumor study group siopel-1 study. J Clin Oncol. (2005) 23(6):1245–52. 10.1200/JCO.2005.07.145 15718322

[12] Hishiki T. Current therapeutic strategies for childhood hepatic tumors: surgical and interventional treatments for hepatoblastoma. Int J Clin Oncol. (2013) 18(6):962–8. 10.1007/s10147-013-0625-7 24132546

[13] Schnater JM Aronson DC Plaschkes J Perilongo G Brown J Otte JB Surgical view of the treatment of patients with hepatoblastoma: results from the first prospective trial of the international society of pediatric oncology liver tumor study group. Cancer. (2002) 94(4):1111–20. 10.1002/cncr.10282 11920482

[14] Czauderna P. Is it worth completely resecting hepatoblastoma at diagnosis? Lancet Oncol. (2019) 20(5):614–5. 10.1016/S1470-2045(19)30096-8 30975628

[15] Czauderna P Garnier H. Hepatoblastoma: current understanding, recent advances, and controversies. F1000Res. (2018) 7:53. 10.12688/f1000research.12239.1 29375822 PMC5770992

[16] Katzenstein HM Langham MR Malogolowkin MH Krailo MD Towbin AJ McCarville MB Minimal adjuvant chemotherapy for children with hepatoblastoma resected at diagnosis (Ahep0731): a children’s oncology group, multicentre, phase 3 trial. Lancet Oncol. (2019) 20(5):719–27. 10.1016/S1470-2045(18)30895-7 30975630 PMC6499702

[17] Moosburner S Schmelzle M Schoning W Kastner A Seika P Globke B Liver transplantation is highly effective in children with irresectable hepatoblastoma. Medicina (Kaunas). (2021) 57(8):819. 10.3390/medicina57080819 34441025 PMC8399470

[18] Douglass EC Reynolds M Finegold M Cantor AB Glicksman A. Cisplatin, vincristine, and fluorouracil therapy for hepatoblastoma: a pediatric oncology group study. J Clin Oncol. (1993) 11(1):96–9. 10.1200/JCO.1993.11.1.96 8380296

[19] Zhang G Zhou XJ Zhu CZ Dong Q Su L. Usefulness of three-dimensional(3d) simulation software in hepatectomy for pediatric hepatoblastoma. Surg Oncol. (2016) 25(3):236–43. 10.1016/j.suronc.2016.05.023 27566028

[20] Shen G Wu L Zhao J Wei B Zhou X Wang F Clinical and pathological study of tumor border invasion-is narrow resection margin acceptable in hepatoblastoma surgery? Front Med (Lausanne). (2020) 7:59. 10.3389/fmed.2020.00059 32195259 PMC7064447

[21] Yuan XJ Wang HM Jiang H Tang MJ Li ZL Zou X Multidisciplinary effort in treating children with hepatoblastoma in China. Cancer Lett. (2016) 375(1):39–46. 10.1016/j.canlet.2016.02.051 26945966

[22] Agarwala S Gupta A Bansal D Vora T Prasad M Arora B Management of hepatoblastoma: ICMR consensus document. Indian J Pediatr. (2017) 84(6):456–64. 10.1007/s12098-017-2301-9 28353129

[23] Hiyama E Hishiki T Watanabe K Ida K Yano M Oue T Mortality and morbidity in primarily resected hepatoblastomas in Japan: experience of the JPLT (Japanese study group for pediatric liver tumor) trials. J Pediatr Surg. (2015) 50(12):2098–101. 10.1016/j.jpedsurg.2015.08.035 26388131

[24] Wang TY Han YL Gao YJ Xu M Gu S Yin MZ Retrospective analysis of childhood hepatoblastoma in a single centre in China. Clin Oncol (R Coll Radiol). (2019) 31(7):471–8. 10.1016/j.clon.2019.03.044 31000431

[25] Zsiros J Brugieres L Brock P Roebuck D Maibach R Zimmermann A Dose-dense cisplatin-based chemotherapy and surgery for children with high-risk hepatoblastoma (siopel-4): a prospective, single-arm, feasibility study. Lancet Oncol. (2013) 14(9):834–42. 10.1016/S1470-2045(13)70272-9 23831416 PMC3730732

[26] Trobaugh-Lotrario AD Katzenstein HM. Chemotherapeutic approaches for newly diagnosed hepatoblastoma: past, present, and future strategies. Pediatr Blood Cancer. (2012) 59(5):809–12. 10.1002/pbc.24219 22648979

[27] Marin JJG Cives-Losada C Asensio M Lozano E Briz O Macias RIR. Mechanisms of anticancer drug resistance in hepatoblastoma. Cancers (Basel). (2019) 11(3):407. 10.3390/cancers11030407 PMC6468761 30909445

[28] Knight KR Chen L Freyer D Aplenc R Bancroft M Bliss B Group-wide, prospective study of ototoxicity assessment in children receiving cisplatin chemotherapy (Accl05c1): a report from the children’s oncology group. J Clin Oncol. (2017) 35(4):440–5. 10.1200/JCO.2016.69.2319 27937095 PMC5455699

[29] Clemens E de Vries AC Am Zehnhoff-Dinnesen A Tissing WJ Loonen JJ Pluijm SF Hearing loss after platinum treatment is irreversible in noncranial irradiated childhood cancer survivors. Pediatr Hematol Oncol. (2017) 34(2):120–9. 10.1080/08880018.2017.1323985 28590156

[30] Dicken BJ Bigam DL Lees GM. Association between surgical margins and long-term outcome in advanced hepatoblastoma. J Pediatr Surg. (2004) 39(5):721–5. 10.1016/j.jpedsurg.2004.01.035 15137006

[31] Ren X Li H Diao M Chen L Xu H Li L. Results of surgical resections with positive margins for children with hepatoblastoma: case series from a single Asian center. Pediatr Blood Cancer. (2019) 66(1):e27479. 10.1002/pbc.27479 30255649

[32] Aronson DC Weeda VB Maibach R Czauderna P Dall’Igna P de Ville de Goyet J Microscopically positive resection margin after hepatoblastoma resection: what is the impact on prognosis? A childhood liver tumours strategy group (siopel) report. Eur J Cancer. (2019) 106:126–32. 10.1016/j.ejca.2018.10.013 30528797

[33] Yoon HM Hwang J Kim KW Namgoong JM Kim DY Koh KN Prognostic factors for event-free survival in pediatric patients with hepatoblastoma based on the 2017 pretext and chic-hs systems. Cancers (Basel). (2019) 11(9):1387. 10.3390/cancers11091387 PMC6769992 31540387

[34] Li F Zhang W Hu H Zhu X Zhang Y Huang D. Factors influencing recurrence after complete remission in children with hepatoblastoma: a 14-year retrospective study in China. PLoS One. (2021) 16(11):e0259503. 10.1371/journal.pone.0259503 34843510 PMC8629180

[35] Qiao GL Li L Cheng W Ge J Zhang Z Wei Y. Predictors of survival after resection of children with hepatoblastoma: a single Asian center experience. Eur J Surg Oncol. (2014) 40(11):1533–9. 10.1016/j.ejso.2014.07.033 25103357

[36] Czauderna P Haeberle B Hiyama E Rangaswami A Krailo M Maibach R The children’s hepatic tumors international collaboration (chic): novel global rare tumor database yields new prognostic factors in hepatoblastoma and becomes a research model. Eur J Cancer. (2016) 52:92–101. 10.1016/j.ejca.2015.09.023 26655560 PMC5141607

[37] Lake CM Tiao GM Bondoc AJ. Surgical management of locally-advanced and metastatic hepatoblastoma. Semin Pediatr Surg. (2019) 28(6):150856. 10.1016/j.sempedsurg.2019.150856 31931965

